# Perspectives on lecithin from egg yolk: Extraction, physicochemical properties, modification, and applications

**DOI:** 10.3389/fnut.2022.1082671

**Published:** 2023-01-06

**Authors:** Feng Zhao, Rongji Li, Yun Liu, Haiyan Chen

**Affiliations:** ^1^College of Food Science and Engineering, Jilin Agriculture University, Changchun, Jilin, China; ^2^College of Life Sciences, Beijing University of Chemical Technology, Beijing, China; ^3^College of Food Science and Engineering, Changchun Sci-Tech University, Changchun, Jilin, China; ^4^College of Pharmacy, Changchun University of Chinese Medicine, Changchun, Jilin, China

**Keywords:** egg yolk lecithin, biological modification, metabolism emulsion, review, food nutrient

## Abstract

Egg yolk lecithin has physiological activities as an antioxidant, antibacterial, anti-inflammatory, and neurologic, cardiovascular, and cerebrovascular protectant. There are several methods for extracting egg yolk lecithin, including solvent extraction and supercritical extraction. However, changes in extraction methods and functional activity of egg yolk lecithin are a matter of debate. In this review we summarized the molecular structure, extraction method, and functional activity of egg yolk lecithin to provide a good reference for the development of egg yolk lecithin products in the future.

## 1. Introduction

Phospholipids refer to phosphorus-containing lipids that were first separated from egg yolk by Gobley in 1844 and named lecithin in Greek (1). The components of egg yolk lecithin include phosphatidylcholine (PC), phosphatidylethanolamine (PE), and lysophosphatidyl choline (2). Phosphatidylcholine (PC) is the main component of lecithin, the content of which is ~73.0%. The content of lecithin in egg yolk is three times higher than the content of lecithin in soybean. It has been shown that the content of lecithin in egg yolk and duck egg yolk is large, accounting for ~10% of total lecithin (3). Phospholipids are one of the basic components of cell membranes.

The membrane acts as a protective barrier for the cell, a channel for the exchange of environmental substances inside and outside the cell. The membrane is the site where many enzyme systems carry out biochemical reactions, which are the basis of living substances. Phospholipids participate in cell metabolism and have unique structures and properties, ensuring the normal functioning of cells (4). Phospholipids are also an important part of brain cells and nerve tissues, accounting for 30% of the weight of the brain, and are very important for the transmission of neuronal information within the brain (5).

Egg yolks and some oil crop seeds (soybeans and rapeseeds) contain the most abundant amount of lecithin. Due to the high cost of preparing lecithin from egg yolks, soybeans are the main source of lecithin. Compared with plant-derived phospholipids, egg yolk phospholipids have a more balanced and unique phospholipid composition (Table 1) (6) and contain specific fatty acids that are not found in plant-derived phospholipids (Table 2) (7). Lecithin derived from egg yolk is a component of the granular part of egg yolk, which accounts for ~70% of all phospholipids in egg yolk (8). Egg yolk lecithin delays aging, protects the stomach and liver, supports the utilization of fat-soluble vitamins, improves the efficiency of blood circulation, and has good physiologic and pharmaceutical functions (9). Egg yolk lecithin is also a basic component of special medicinal emulsions and has the potential to become a new generation of drugs. At present, egg yolk lecithin has been used to improve memory in schizophrenia, childhood autism, and Alzheimer's disease, and as an anti-oxidant during organ transplantation (10).

**Table 1:** Phospholipid composition of egg yolk.

**Component**	**Content** **%**	**Component**	**Content** **%**
Phosphocholine	73.0	Hemolyso phosphorylcholine	5.8
Phosphoethanolamine	15.0	Hemolytic phosphoethanolamine	2.1
Phosphoacylserine	0.9	Sphingomyelin	2.5
Phosphatidylinositol	0.6	Other phospholipids	0.1

**Table 2:** Fatty acid component and content of yolk phospholipid.

**Component**	**Content%**	**Component**	**Content%**
Myristic acid (C14:0)	0.23	Oleic acid (C18:1)	57.80
Pentadecanoic acid (C15:0)	0.10	Linoleic acid (C18:2)	7.45
Palmitic acid (C16:0)	19.44	Linolenic acid (C18:3)	1.67
Palmitic acid (C16:1)	1.09	Arachidonic acid (C20:4)	0.83
Heptadecanoic acid (C17:0)	0.33	Docosahexaenoic acid (C22:6)	2.62
Stearic acid (C18:0)	7.72	Other	0.72

## 2. Method for extracting egg yolk lecithin

### 2.1. Organic solvent extraction

**Figure 1:**
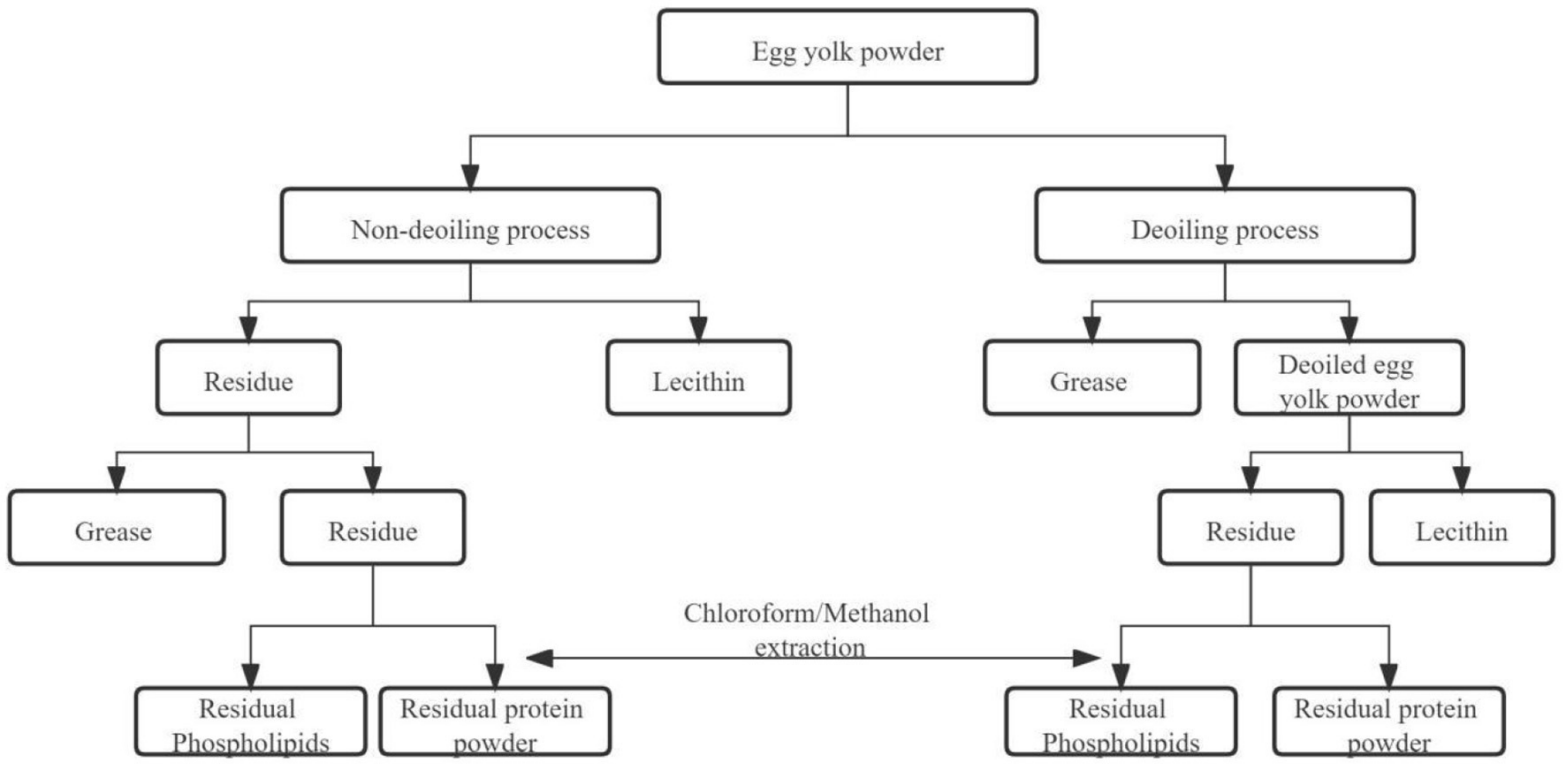
Process of leclithin by drying egg yolk powder as a raw solvent.

Solvent extraction is a method to separate and extract lecithin by using the difference in the selectivity of the solvent to the components of phospholipids. Solvent extraction is also the earliest method used for phospholipid extraction (11). There are two methods (single and mixed organic solvent extraction methods). During single organic solvent extraction, ethanol is used to extract lecithin from fresh egg yolk and the extraction rate can be 93.38% under suitable process conditions (12). The mixed organic solvent extraction method uses the characteristics of lecithin that is soluble in ethanol, but insoluble in acetone to extract lecithin from eggs. The organic solvent extraction method is characterized as a simple operation and low requirements for conditions, but the organic solvent extraction method takes too long, the purity is not high, and there may be residual organic solvents (Figure 1).

During the actual operation process, to improve the extraction efficiency some new auxiliary technologies have been developed to facilitate the extraction. For example, the effect of the electric pulse generated by the high-voltage pulse electric field on lecithin is assisted by the organic solvent method, and the extraction rate of egg yolk lecithin is increased by 10.2% compared with the traditional method (13). The high-voltage pulsed electric field-assisted method has the advantages of safety, speed, efficiency, and less damage to nutrients. Using ultrasonic- or microwave-assisted separation (14), ultrasonic-assisted separation can accelerate the rupture of the cell wall, and make the extracted substance and the extraction solvent more accessible.

### 2.2. Super- and sub-critical extraction

**Figure 2:**
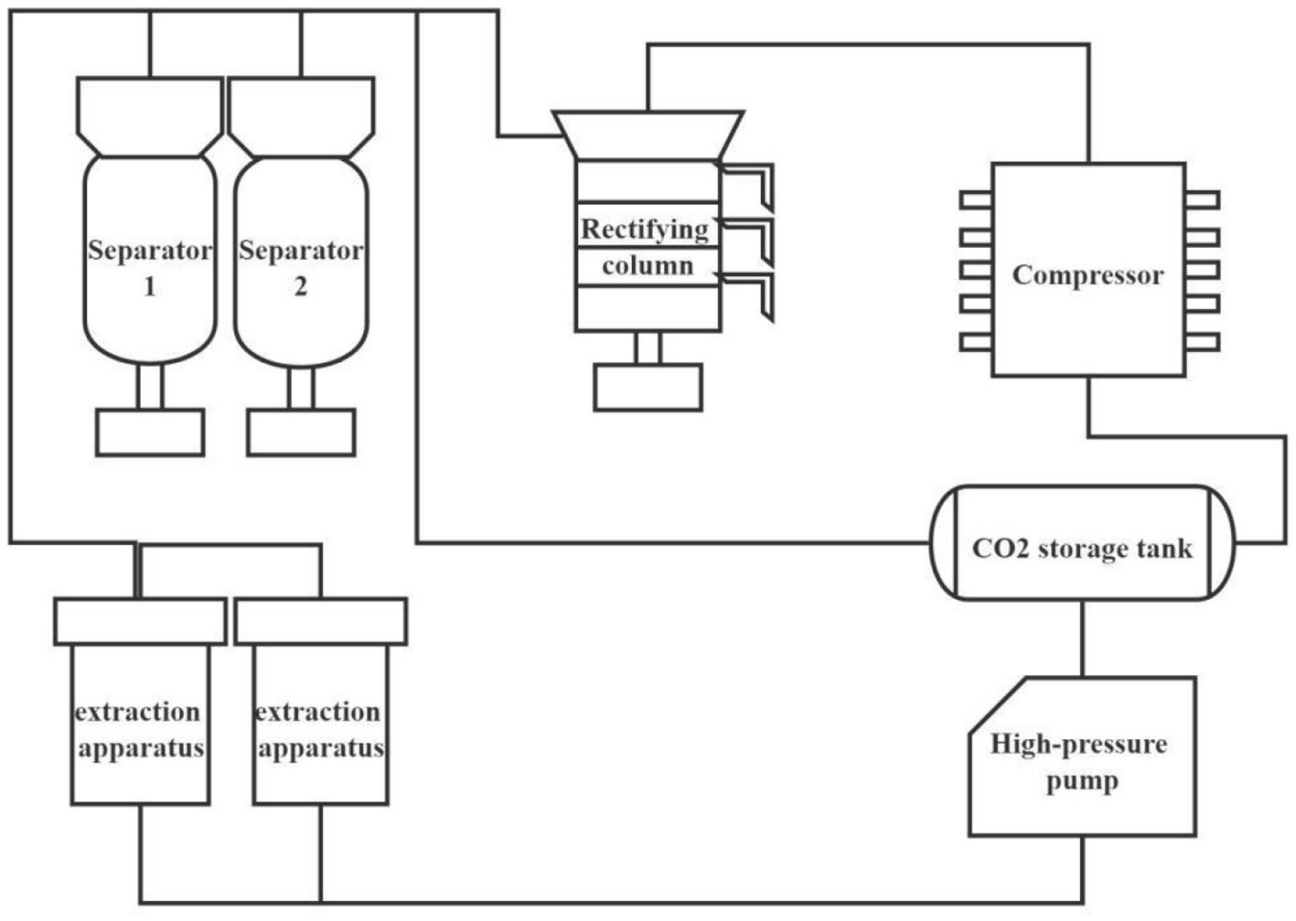
Supercritical extraction equipment.

Supercritical extraction is a new type of separation technology that separates substances by changing the temperature and pressure according to the difference in the properties of substances in the supercritical state. Supercritical carbon dioxide extraction technology can extract lecithin with higher purity (95–98%) from egg yolk powder (15). Because phospholipids are insoluble in supercritical CO_2_, it is necessary to add an entrainer to grade phospholipids (Figure 2) (16).

Subcritical extraction refers to a fluid extraction technology that uses a thermodynamic state at the edge of a supercritical state and is above the critical pressure and below the critical temperature. Compared with the supercritical fluid method, the subcritical fluid method has milder conditions and less stringent requirements for high-pressure equipment. And on the other hand, the subcritical fluid method can better retain the natural active ingredients in the extracted product with a lower cost.

### 2.3. Enzymatic hydrolysis extraction method

The enzymatic hydrolysis method uses proteases to break large molecular proteins into small molecular peptides, speed up the separation of lecithin and protein, shorten the extraction process of lecithin, thus improve the extraction efficiency of egg yolk lecithin. The volume fraction of ethanol is 95% and the volume fraction of protease is 0.06%. The extraction efficiency of lecithin is higher when performed for 2 h at 35°C (17). The enzymatic hydrolysis method has a mild operating environment and can obtain relatively high-quality proteins and lipids. To further improve the extraction efficiency, ultrasonic-assisted enzymatic extraction can be used (18).

### 2.4. Column lamination method

Column chromatography uses an adsorbent as a stationary phase. When the solute in the mobile phase passes through the stationary phase, the solute achieves the purpose of separation due to different adsorption and resolution capabilities. Currently, silica gel and alumina ion exchange resin are commonly used as stationary phases in column chromatography. Moreover, the main method for producing high-purity egg yolk lecithin is column chromatography but column chromatography requires an adsorption-desorption process, the processing volume is small, and the solvent consumption is large. To avoid the residual toxicity of the solvent, the less toxic ethanol is generally used as the mobile phase (19).

### 2.5. Other extraction methods

In addition to the above-described methods, several more common methods of extracting lecithin exist, such as the cryo-precipitation (14) and membrane separation methods (20). Among them extraction methods, membrane technology is a relatively new technology for separating substance mixtures. Natural or synthetic membranes are used to provide driving force in external energy or chemical potential differences (pressure difference, concentration difference, and potential difference). Under such conditions, the raw material side components selectively permeate the membrane to achieve the technical method of sample separation, classification, enrichment, and purification. Hollow fiber ultrafiltration membranes and organic polymer membranes have been reported for phospholipid separation (21).

## 3. Basic properties of egg yolk lecithin

### 3.1. Physical and chemical properties of egg yolk lecithin

High-purity egg yolk lecithin products are white, waxy solids. When lecithin is in a liquid form, it is light yellow and slippery with a peculiar smell. Egg yolk lecithin contains an abundance of unsaturated fatty acids, most of lecithin products appear slightly darker than they are supposed to, due to the unsaturated fats being oxidized. In the production of lecithin, the dosage forms are in different forms due to the concentration. The concentration of liquid is ~60%, and the concentration of granules and powder can reach more than 95%.

Egg yolk lecithin is a natural phospholipid mixture extracted and refined from egg yolk, and is an amphiphilic molecule. According to different types of backbone alcohols, egg yolk lecithins are mainly divided into two categories: based on glycerol (glycerophospholipids) and on sphingosine (sphingomyelin). Each 100 g of egg yolk contains 9.44 g of phospholipids, 1,011 mg of cholesterol, 0.83 mg of lutein, 0.42 mg of zeaxanthin, 0.53 mg of canthaxanthin, and 0.11 mg of β-carotene (22). The volume of egg yolk is 30–32% of a whole egg. The whole egg contains fat (30%), protein (15%), moisture (50%), and other chemicals. Gazolu-Rusanova et al. (23) used SDS-PAGE to isolate the type and composition of egg yolk protein. After freezing and centrifugation, the yolk was divided into two parts: supernatant; and sediment. The structure of the supernatant part is mainly in the form of aggregates, and the precipitation part is mainly spherical. The supernatant accounts for 77–81% of the dry matter weight of the egg yolk, and the sediment accounts for 19–23%. The supernatant contains 85% low-density lipoprotein (LDL) and 15% egg yolk protein (livetins).

The precipitate contains 70% high-density lipoprotein (HDL), 16% high phosphoprotein (phosvitin), and 12% low-density lipoprotein (LDL) (24). The structural features of glycerol lecithin are as follows: hydroxyl groups on glycerol sn-1 and sn-2 are esterified by saturated or unsaturated fatty acids; the hydroxyl groups on sn-3 are esterified by phosphoric acid; and the phosphoric acid is connected to the base according to the main types of glycerol lecithin, including PC, phosphatidylethanolamine (PE), phosphatidylinositol (PI), phosphatidylserine (PS), and phospholipids, phosphatidic acid (PA), and phosphatidylglycerol (PG).

In addition to the above six types of glycerol lecithin, the fatty acyl group at the sn-1 position of glycerol in the glycerophospholipid molecule is replaced by a long-chain alcohol to form a vinyl ether, which is referred to as plasmalogen. The phosphoryl group in the glycerophospholipid molecule is replaced by a phosphate group, which is referred to as a phosphate ester. Using phospholipase and specific lipase to hydrolyze glycerol lecithin produces lysophosphatidylcholine. Gazolu-Rusanova et al. (23) showed that the lysophosphatidylcholine oil/water interface and liquid membrane properties have a very important role. Sphingomyelin is composed of sphingosine, a fatty acid, phosphoric acid, and nitrogenous bases. The fatty acyl and cerol amino groups are connected by an amide bond, and the sphingosine that is formed is also referred to as ceramide. The primary alcohol group of cerol is connected to phosphatidylcholine or phosphatidylethanolamine by a phosphate bond. The fatty acids found in sphingomyelin include palmitic acid, stearic acid, tar oleic acid, and cerene acid.

### 3.2. Physiological function of egg yolk lecithin

#### 3.2.1. Strengthening nerve conduction

Brain nerve cells contain a large amount of lecithin. The content of lecithin accounts for approximately one-fifth of brain nerve cell mass. Lecithin in brain cells is transformed to release choline, which combines with acetyl-CoA to produce acetylcholine. Acetylcholine is an important chemical transmitter for information transmission between various nervous systems; so lecithin can increase the degree of brain cell activation, and improve memory and intelligence. Using the brain for a long time in life and work will consume a large amount of lecithin in the body, leading to a decline in brain function. Relevant studies have shown that with age, brain function gradually declines. Consuming lecithin products effectively prevents further aging of the brain and strengthens brain function. During the growth stage, children should eat foods rich in lecithin nutrients, such as eggs. Nuts and animal liver have a positive effect on brain development, learning, and memory as well.

Alzheimer's disease is a neurodegenerative disease characterized by a loss of memory and cognitive impairment. Eating egg yolk lecithin improves memory and cognitive function (25), and delays the onset of neurodegenerative disease. Specifically, the unsaturated fatty acid chain in egg yolk lecithin structure has an indirect protective role (26). Egg yolk lecithin inhibits acetylcholinesterase activity, reduces the concentration of oxidation products, and exerts a neuroprotective function (27, 28).

#### 3.2.2. Regulation of blood lipids

In recent years, hyperlipidemia has become a common metabolic disorder, causing cardiovascular and cerebrovascular diseases (29) that negatively impact healthful living. The molecular structure of egg yolk lecithin has dual characteristics (hydrophilicity and lipophilicity). Therefore, an emulsification reaction occurs in the body, combining lipid substances with water and emulsifying the accumulated cholesterol and neutral fats in the blood vessels. Egg yolk lecithin serves as some kind of “garbage” (i.e., cholesterol and neutral fats) remover in blood vessels, making blood circulation in the blood vessels of the entire body smoother, promoting blood flow, and penetration into the body, thereby reducing the viscosity of human blood. Thus, egg yolk lecithin can effectively reduce hyperlipidemia and coronary heart disease. Epidemiologic studies have shown that hypertension is a major cause of heart-blood diseases, such as sudden death and coronary heart disease. Egg yolk lecithin lowers blood pressure (28) by inhibiting angiotensin-converting enzyme (ACE.) Skorkowska-Telichowska (30) reported that 15 mL of lecithin fed to patients with metabolic syndrome three times a day significantly improved symptoms. Moreover, the daily intake of 1.2 mmol/L of egg yolk lecithin inhibited cholesterol absorption and transport, and prevented obesity (7, 31).

#### 3.2.3. Repair biofilms and delay body aging

Lecithin is very important for the composition of animal somatic cells. Without lecithin, cell membranes will be damaged to varying degrees. Lecithin is an indispensable substance in cells. PC promotes the synthesis and regeneration of lipoproteins (32), repairs damaged cell membranes, increases desaturation of cell membrane fatty acids, softens and rejuvenates the cell membrane, protects mitochondria and microsomal membranes of somatic cells, and maintains cell structure. In the process of individual aging, the function of the antioxidant defense system in the body gradually weakens, and free radicals accumulate, which will cause excess free radicals to react with unsaturated fatty acids to form a peroxide and eventually deform the organelles through a series of interactions in which neurons are damaged and lecithin has the ability to scavenge free radicals (33), which improves the metabolic capacity. Lecithin can also promote skin regeneration, make the skin shiny, and prevent hair loss, which makes gray hair darker and slows the aging process.

#### 3.2.4. Human nutritional needs

Lecithin provides 90% of the exogenous choline needed by the human body. Lecithin provides two main benefits for choline. First, unlike bound choline, free choline is degraded to methylamine by intestinal microorganisms. Second, choline is obtained by continuous methylation of phosphatidylcholine in the liver and other fibrous tissues, and the synthesis process requires time. Therefore, when dietary choline is insufficient, the endogenous resources of lecithin can supplement body demands.

#### 3.2.5. Egg yolk lecithin liposome

**Figure 3:**
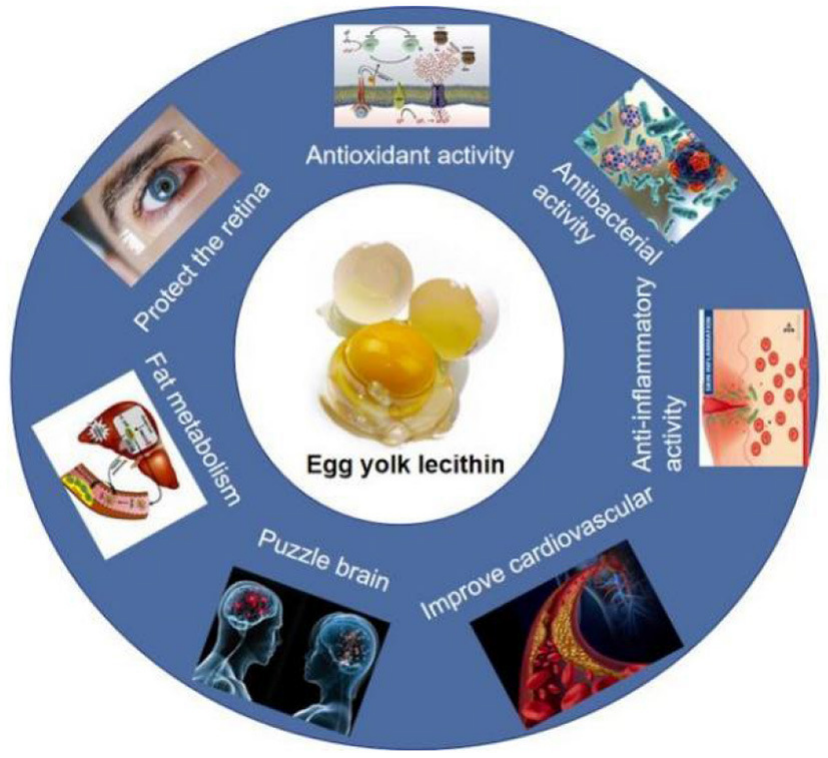
Physiologic function activity of egg yolk lecithin.

Egg yolk lecithin liposomes are drug carriers. With distinct targeting, egg yolk lecithin liposomes are an important preparation in the drug delivery system. Fatty acid composition and species of egg yolk lecithin have a great influence on liposome properties. Saturated fatty acids in the lecithin structure enhance the firmness and non-permeability of the liposome membrane. Unsaturated fatty acids in lecithin structures make liposomes have a lower phase transition, good fluidity, and low viscosity. Four types of egg yolk lecithin liposomes, which are conventional liposomes (34–36), PEG-modified liposomes (37–39), multifunctional liposomes (40–42), and ligand-targeted liposomes (43–45) currently exist. Each liposome has its own advantages and application fields. Five yolk lecithin liposomes were prepared by Kondratowicz (46), who compared the structural and mechanical properties. Both the main components of liposomes (e.g., lecithin, glycerin, and cholesterol) and the component proportions influence the structure of liposomes. Trace components, such as tocopherol and carotene, also have a great impact (see Figure 3).

## 4. Application of yolk lecithin

The present commercial egg yolk lecithin is mainly used in cosmetics, medications, and nutrition (47), and can be used in the preparation of liposomes (48) and fat emulsions. Liposomes are a new preparation and research focusing on drug carriers in a drug release system, and it has become one of the main directions of the manufacturing industry (49). The performance of liposomes from egg yolk, soybean, and porcine lecithin have been compared (50); the results showed that liposomes made of egg yolk lecithin had the best performance. Fat milk injection is a type of nutritional injection used in the clinical setting. Fat milk is an energy supplement outside the gastrointestinal tract. Fat milk can supplement essential fatty acids and energy for patients, and egg yolk lecithin is used as an important component emulsifier. Because lecithin is an important component of biofilms, it can also be used as a drug carrier to form complexes (51) with other drug components, which can directly transport drug components to a diseased site and improve bioavailability.

Egg yolk lecithin is widely used in the feed industry and has significant effects (52). Adding phospholipids to broiler feed improves growth, increases storage in the liver, and promotes bone growth. The different growth periods of fish and shrimp also require the addition of lecithin in feed (46). Other egg yolk lecithin can also be used as a fungicide for crops, a preservative for fruits and flowers, ink emulsifiers, and petroleum products (53).

## 5. Prospects of egg yolk lecithin

Lecithin is not only a high nutritional value substance, but also a highly bioactive functional component (54). For >30 years, extensive and in-depth research has been conducted on transmitters of sustained-release drugs, maintenance of function, development of functional foods, and means to improve industrial production (2). As a natural emulsifier and wetting agent, lecithin, in addition to its biological efficacy, is often used as an emulsifier for intravenous fat injection and is the main embedding material for liposomes (55). Lecithin has unique membrane permeability, and it is of high value to the packaging industry (56). At present, with the continuous improvement in the lecithin extraction and preparation process, lecithin with a high purity, low price, pure nature, and no side effects will be fully utilized and developed (57). At the same time, with the development of lecithin, a new direction for deep processing of eggs will emerge (58).

Currently, high-purity lecithin is in high demand in the international market (59). High-purity lecithin products usually refer to phospholipid products containing ~95% PC (60). Because high-purity lecithin products are pure, have no peculiar smell, strong emulsification, and are easy to dissolve in water, they can be added and used in large quantities in the food industry and can also be made into health products and pharmaceuticals (13). The price of phospholipid products is ten times or even dozens of times higher than crude lecithin (61). Phospholipid production in China is far behind foreign countries in terms of its production scale and technical level (62). Medical oral liquid and high-purity phospholipids for injection still need to be imported in large quantities (13). With regards to deep processing of agricultural products, the production of lecithin is currently one of the key technical development areas supported by the State (63). Therefore, the prospect of developing lecithin and its deep-processed products in China is very broad.

## 6. Conclusions

The extraction method of lecithin in eggs is mainly based on the extraction method of soybean phospholipids, organic solvent extraction, supercritical fluid extraction, and column chromatography. With the development of technology, the enzymatic method and membrane separation method will make the extraction of lecithin from eggs more convenient, fast, efficient and environmentally-friendly. The author believes that the basic physical and chemical properties of egg yolk lecithin, the optimization of extraction methods, technological innovation, and the development of functional properties should be studied in depth to explore differences and characteristics superior to soy lecithin to develop research methods and production suitable for egg yolk lecithin. Application technology provides theoretical basis and technical support for the future application of egg yolk lecithin in various fields.

## Author contributions

Guarantor of integrity of the entire study and manuscript preparation: HC. Study concepts, study design, literature research, data analysis, and manuscript editing: FZ. Definition of intellectual content: HC and YL. Clinical studies and statistical analysis: RL. Data acquisition and manuscript review: YL. All authors contributed to the article and approved the submitted version.
